# Supplementary material for: Myelo-lymphoid lineage restriction occurs in the human haematopoietic stem cell compartment before lymphoid-primed multipotent progenitors
Source: Nat Commun. 2018 Oct 5;9:4100. doi: 10.1038/s41467-018-06442-4 (PMC6173731; doi:10.1038/s41467-018-06442-4)
Supplement: Supplementary file 1 — Supplementary Information [file 41467_2018_6442_MOESM1_ESM.pdf]

SUPPLEMENTARY INFORMATION:

Myelo-lymphoid lineage restriction occurs in the human haematopoietic stem cell compartment before lymphoid-primed multipotent progenitors

Belluschi et al.

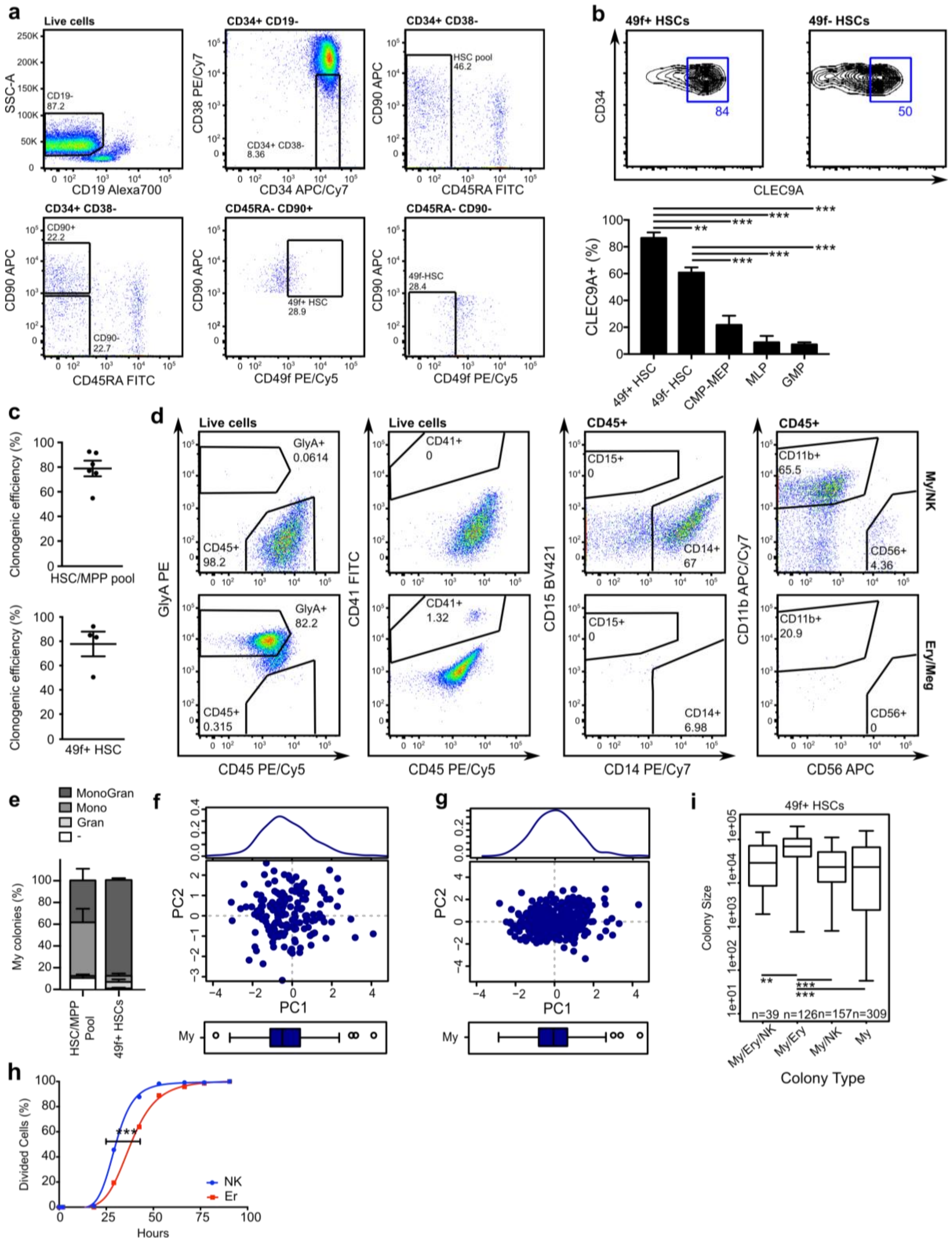

Supplementary Figure 1: Sorting strategies and analysis of single cell derived colonies.

a, Representative example of gating strategy used to isolate HSC/MPPs (Fig. 1a-c, Fig. 1h, Fig. 2a), 49f^{+} HSCs (Fig. 1d-g, Fig. 1i-k, Fig.2a. Fig. 3a-b, Fig. 4a) and 49f^{-} HSCs from

$CD34^+$ CB cells. $49f^+$ HSCs are defined as the highest 30% $CD90^+$ $CD49f^+$, and $49f^-$ HSCs as the lowest 30% $CD90^-$ $CD49f^-$ [1]. **b,** Representative example of CLEC9A cell surface expression in CB $49f^+$ HSCs (top left, count=307 cells) and $49f^-$ HSCs (top right, count=296 cells). Percentage of $CLEC9A^+$ cells in the indicated populations (lower panel). $^{**}p<0.01$, $^{***}p<0.001$ by one-way ANOVA with Tukey's multiple comparison. Mean ± SEM is shown. **c,** Clonogenic efficiency of single HSC/MPP pool cells (n=537 single cells plated from 6 independent CBs; top panel) and single $49f^+$ HSCs (n=1104 single cells plated from 4 independent CBs; bottom panel) in the single cell differentiation assay. Mean ± SEM is shown. **d,** Gating strategy used for lineage determination of colonies derived from HSC/MPP pool or $49f^+$ HSC single cells. Erythroid colonies were identified as $GlyA^+ \geq 30$ cells, megakaryocytic colonies as $CD41^+ \geq 30$ cells, myeloid colonies as $[(CD45^+\ CD14^+) + (CD45^+\ CD15^+)] \geq 30$ cells, NK colonies as $CD45^+\ CD56^+ \geq 30$ cells. Two representative examples (one My/NK and one Ery/Meg colony) are shown. This gating strategy was used for Fig. 1a-h, Fig. 2b-e, Fig. 4c. **e,** Percentage of My colonies of the indicated type MonoGran, Mono, Gran and undetermined (-) derived from HSC/MPP pool single cells (n=362 colonies from 6 independent CBs) and $49f^+$ HSCs (n=725 colonies from 4 independent CBs); Mean ± SEM is shown. **f, g,** Principal Component Analysis (PCA) of cell surface marker expression at the time of sort of single HSC/MPP pool cells isolated from 6 independent CBs (**f**) and single $49f^+$ HSCs isolated from 4 independent CBs (**g**) that produced My colonies (**f,** n=158 cells, **g,** n=309 cells). Density plots (top panel) and boxplots (bottom panel) of PC1. **h,** Representative example of non-linear fit of cumulative first division kinetics for single $49f^+$ HSCs producing colonies containing one of the two indicated lineages (Ery n=91, NK n=139); $^{***}p<0.001$ by extra sum-of-square F test. **i,** Size of different types of colonies (number of cells) derived from $49f^+$ HSC single cells from 4 independent CBs. $^{**}p<0.01$, $^{***}p<0.001$ by Kruskal-

Wallis test with multiple comparison. All boxplots show median, interquartile and 5-95 percentile.

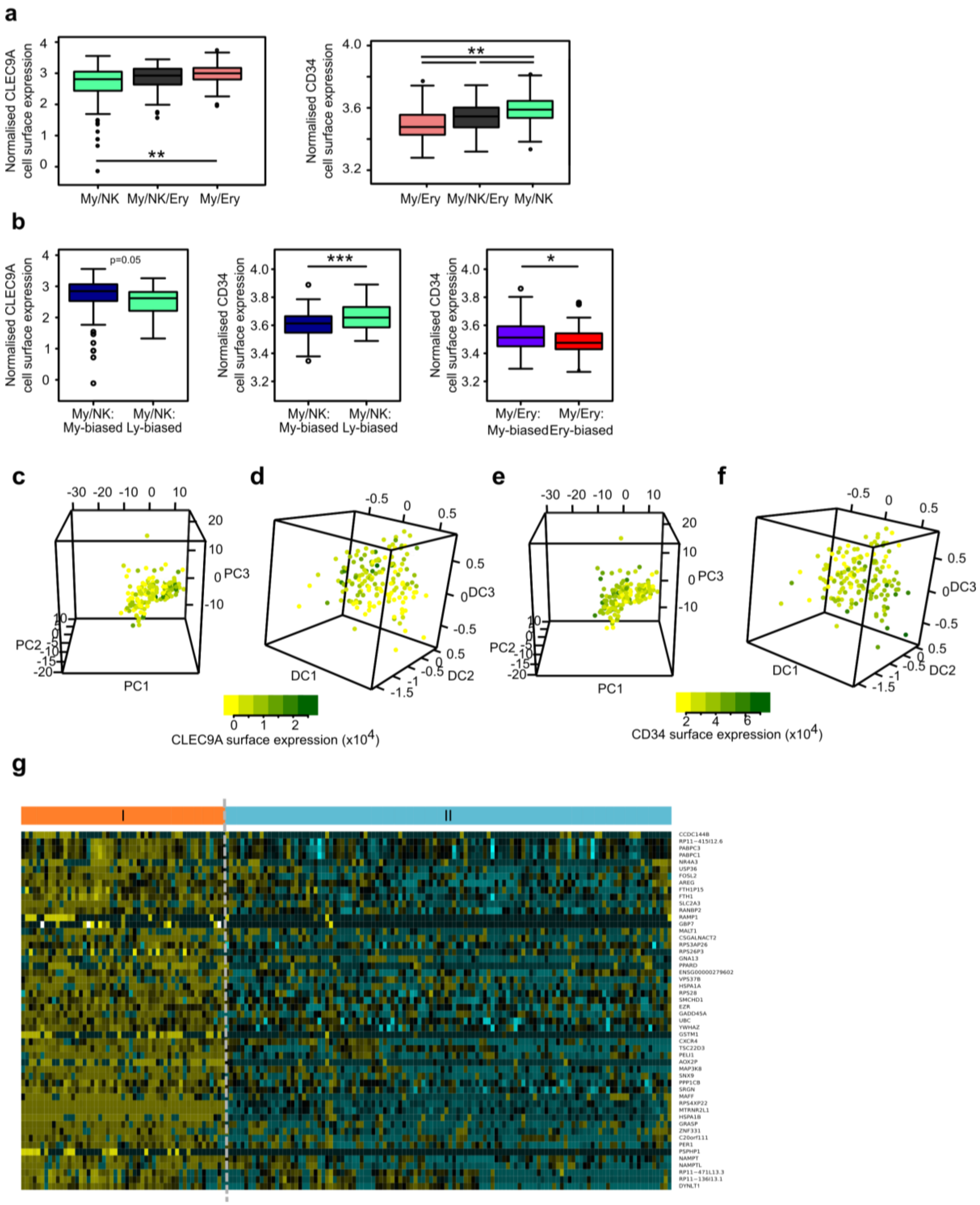

Supplementary Figure 2: Expression of CLEC9A and CD34 polarises the 49f^{+} HSC compartment

a, Normalised intensity of CLEC9A and CD34 expression at the time of the sort for 49f^{+} single cells which produced the indicated colonies. n=714 colonies from 4 independent CBs. Median fluorescence intensity shift between My/Ery and My/NK: CLEC9A: 40% and CD34:

13%. **p<0.01by Kruskal-Wallis test with multiple comparison. **b,** Normalised intensity of CLEC9A and CD34 expression at the time of the sort for 49f$^+$ HSC single cells which produced the indicated colonies. My/NK colonies are defined My-biased if number of My cells > number of Ly (NK) cells within that colony and Ly-biased if number of My cells < number of Ly cells. Similarly, in Ery-biased My/Ery colonies, the number of My cells < number of Ery cells. (n=93 My/Ery colonies and n=170 My/Ly colonies from 4 independent CBs). Median fluorescence intensity shifts: between My/NK: My-biased and My/NK: Ly-biased: CLEC9A: 25%; CD34: 4%; between My/Ery: My-biased and My/Ery: Ery-biased: CD34: 4%. *p<0.05 ***p<0.001 by two-sided unpaired t-test. All boxplots show median, interquartile and 5-95 percentile. **c,** CLEC9A cell surface marker expression overlaid on 3D PCA (**c**) or diffusion map (**d**) representation of 49f$^+$ HSCs. **e, f,** CD34 cell surface marker expression overlaid on 3D PCA (**e**) or diffusion map (**f**) representation of 49f$^+$ HSCs. **g,** Heatmap of expression of guide genes selected by ICGS algorithm performed on 49f$^+$ HSCs. Rows represent genes and columns single cells. Top panel bar shows major clusters identified by ICGS. **c-g:** n=169 single cells that passed quality control.

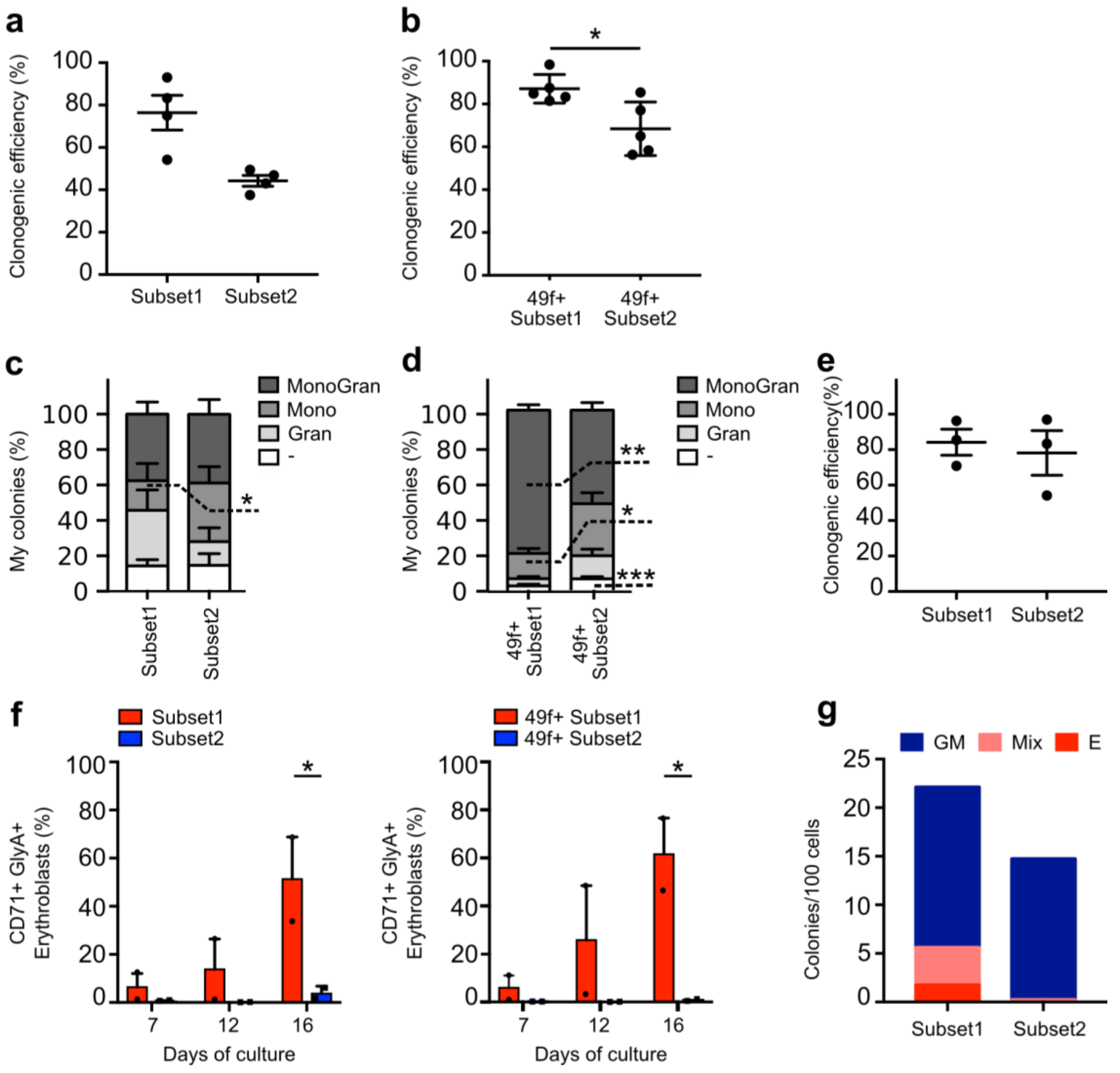

Supplementary Figure 3: Characterisation of Subset1, Subset2, 49f+ Subset1 and 49f+ Subset2 populations.

a, b, Clonogenic efficiency of Subset1 and Subset2 cells (n=4 independent CBs, total 336 cells plated per subset, minimum 72 cells per CB) (**a**), 49f+ Subset1 and 49f+ Subset2 cells (n= 5 independent CBS, 714 total cells plated for 49f+ Subset1 and 756 for 49f+ Subset2, minimum 54 cells per CB, *p<0.05 by two-sided paired t-test) (**b**) in the same single cell differentiation assay as in Fig. 1 a-d. **c, d,** Percentage of My colonies of the type MonoGran, Mono, Gran and undetermined (-) derived from Subset1 (n=194) and Subset2 (n=114) (**c**), 49f+ Subset1 (n=550) and 49f+ Subset2 (n=450) (**d**) single cells from 4 (Subset1, Subset2)

and 5 (49f$^+$ Subset1, 49f$^+$ Subset2) independent CBs; *p<0.05, **p<0.01, ***p<0.001 by two-sided unpaired t-test. **e,** Clonogenic efficiency of Subset1 and Subset2 cells (n=3 independent CBs, total 174 cells plated per subset, minimum 27 cells per CB) in the single cell assay allowing for growth of B, NK and My cells. **f,** Percentage of CD71$^+$GlyA$^+$ erythroblasts obtained at day 7, 12 and 16 by RBC assay after plating 1500 Subset1 and Subset2 cells (left), and 350-1200 cells 49f$^+$ Subset1 and 49f$^+$ Subset2 (right, n=2); n=2 independent CBs; *p<0.05 by 2-way ANOVA with Sidak's multiple comparisons. **a-f,** mean ± SEM is shown. **g,** Number of colonies/100 cells from either Subset1 or Subset2 populations sorted using alternative CD34 and CLEC9A antibody clones and/or fluorochrome combinations (Antibody panel I, see methods) and plated in CFU assay. Colony types: Ery (E), granulocyte and My (GM) or a combination of both (mix) is shown, (n=1 CB).

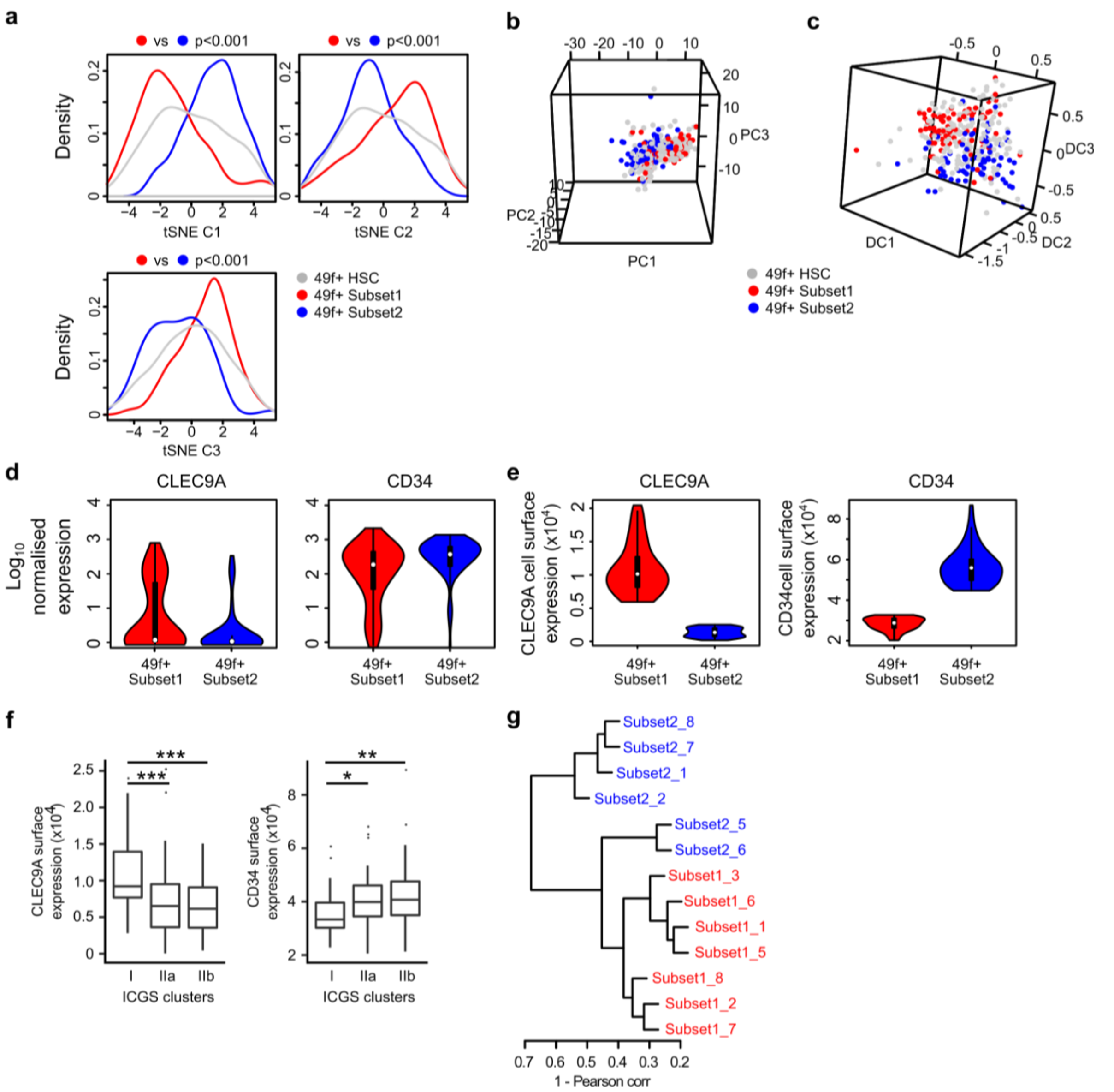

Supplementary Figure 4: Transcriptional landscapes of 49f$^+$ HSCs and Subset1 and Subset2 cells

a-d, Single-cell RNA-seq analysis of 49f$^+$ HSCs (n=169), 49f$^+$ Subset1 (n=78) and 49f$^+$ Subset2 (n=75) single cells. **a,** Density plots of the distribution of single 49f$^+$ HSCs (grey), 49f$^+$ Subset1 (red) and 49f$^+$ Subset2 (blue) cells along the indicated tSNE components. p<0.001 by two-tailed unpaired t-test comparing 49f$^+$ Subset1 and 49f$^+$ Subset2. **b, c,** 3D PCA (**b**) and diffusion map (**c**) representation of single 49f$^+$ HSCs (grey), 49f$^+$ Subset1 (red) and 49f$^+$ Subset2 (blue) cells. All PCA and diffusion map analyses were performed on highly variable genes (2420 genes computed as in [2]). **d,** Log_{10} normalised expression of CLEC9A

(left) and CD34 (right) from 78 $49f^+$ Subset1 single cells and 75 $49f^+$ Subset2 single cells as measured by single cell RNA-seq. **e,** Cell surface expression of CLEC9A (left) and CD34 (right) for the same single cells shown in a as measured by index sorting. **f,** ICGS analysis: cell surface expression levels of CLEC9A (left panel) and CD34 (right panel) in $49f^+$ HSCs of the indicated ICGS clusters (excluding $49f^+$ Subset1 and $49f^+$ Subset2 cells); *p<0.05, **p<0.01, ***p<0.001 by one-way ANOVA with Bonferroni correction for multiple comparisons. Boxplots show median, interquartile and 5-95 percentile. **g,** Hierarchical clustering of highly variable genes of 20-cell samples of Subset1 and Subset2 cells; shown is 1-Pearson correlation; Subset1: n=7; Subset2: n=6 independent 20-cell samples.

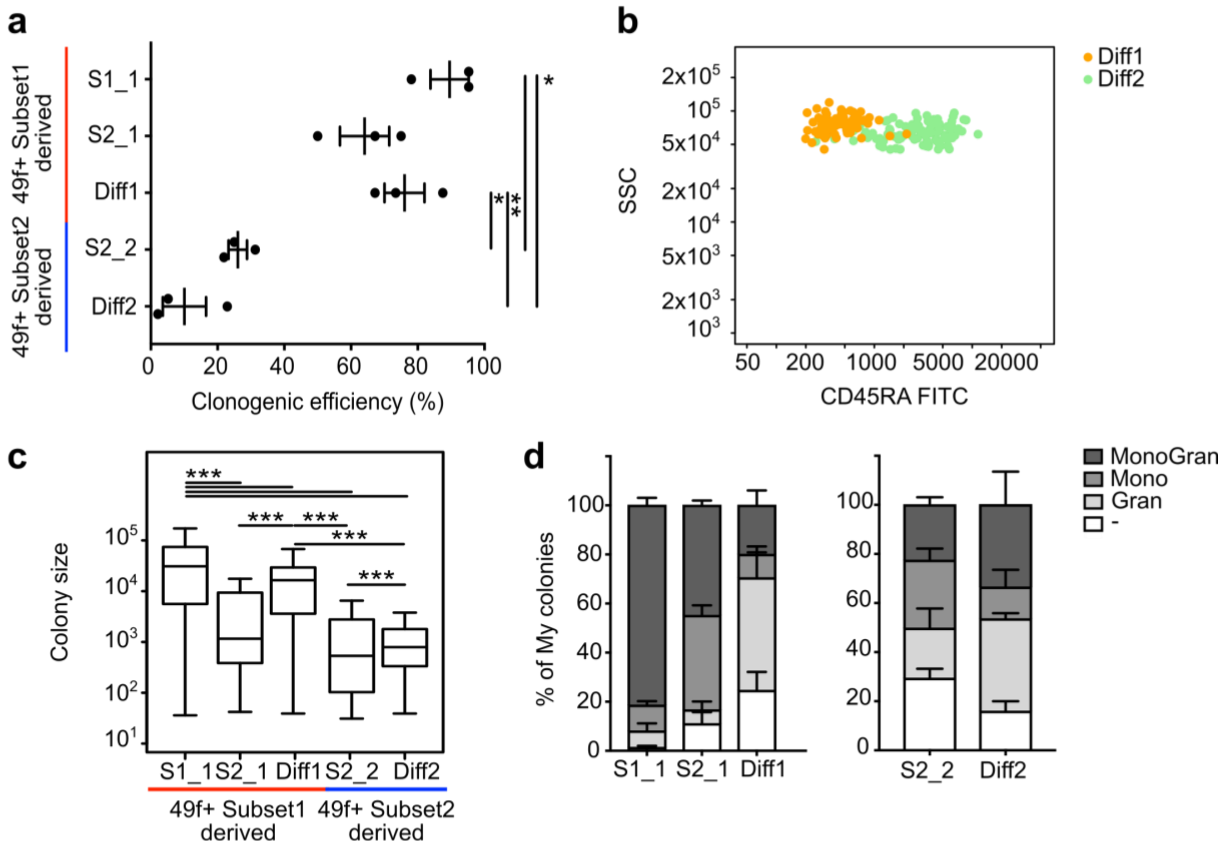

Supplementary Figure 5: Comparison of 49f$^+$ Subset1 and 49f$^+$ Subset2 *in vitro* derived populations.

a, Clonogenic efficiency (calculated as number of colonies/number of seeded cells) of the indicated day 5 populations; mean ± SEM is shown. n=576 cells plated from 3 independent CBs for 49f$^+$ Subset1 derived populations, n=576 cells plated from 3 independent CBs for 49f$^+$ Subset2 derived populations; *p<0.05, **p<0.01 by one way-ANOVA with Tukey's multiple comparison. **b**, CD45RA cell surface intensities for single cells from Diff1 and Diff2 populations (gated as in Fig. 4b) from one representative experiment. **c,** Size of colonies (number of cells) generated by single cells from the indicated 49f$^+$ Subset1 and 49f$^+$ Subset2 derived populations. ***p<0.001 by multiple comparison Kruskal-Wallis test. Boxplots show median, interquartile and 5-95 percentile. **d**, Percentage of My colonies of the MonoGran, Mono, Gran and undetermined types derived from S1_1 (n=159), S2_1 (n=103) and

Diff1(n=72); S2_2 (n=62) and Diff2 (n=22) single cells from 3 independent CBs; mean ± SEM is shown.

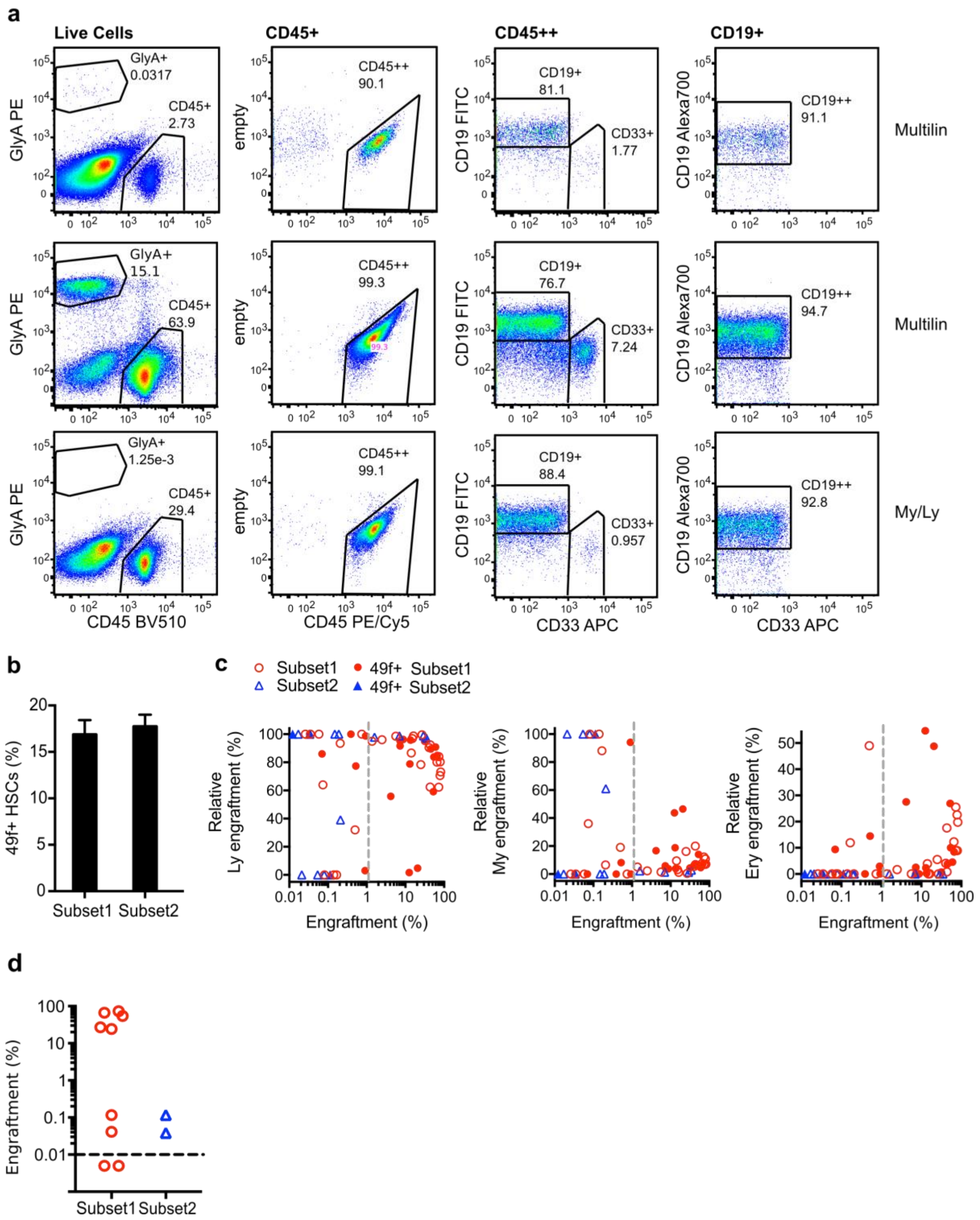

Supplementary Figure 6: Differentiation capacity of Subset1 and Subset2 cells *in vivo*.

a, Representative example of the gating strategy used to assess lineage potential *in vivo*. Ery cells were identified as $CD45^{-}GlyA^{+}$, B cells as $CD45^{++}CD19^{++}$ and myeloid cells as $CD45^{++}CD33^{+}$. To avoid false positives a double staining for CD45 and CD19 was

performed using two different antibodies for each marker (Antibody panel F or G, see methods). Examples of two multi-lineage (My/Ly/Ery) engrafted mice (top and middle) and one My/Ly engrafted mouse (bottom) are shown. These gates were used for the results in Fig. 5. **b,** Percentage of phenotypic $49f^+$ HSCs within Subset1 and Subset2 (n=5 independent CBs), mean ± SEM is shown. **c**, Relative Ly (left), My (middle) and Ery (right) engraftment (percentage of total human grafted cells) as a function of human engraftment (percentage of $CD45^+$ and $GlyA^+$ present in the injected bone of each transplanted mouse). Subset1: n=27 mice; Subset2: n=12 mice; 49f+ Subset1: n=19 mice; $49f^+$ Subset2: n=3 mice. Of note, 13/15 animals engrafted with Subset1 at >1% produced Ery cells, while 0/3 animals transplanted with Subset2 did (p=0.012 by two-tailed Fisher test). **d**, Percentage of human engraftment (% $CD45^+$ + $GlyA^+$) in the injected femur of secondary transplantation recipients injected with $CD34^+CD38^-$ cells isolated from primary mice engrafted with Subset1 (n=9, red) and Subset2 (n=2, blue) cells (see Fig. 5 for primary transplantation data). Dashed line: threshold of engraftment ((%$CD45^{++}$ + %$GlyA^+$)≥0.01% and at least 30 cells recorded. Non-engrafted mice are shown below the dashed line.

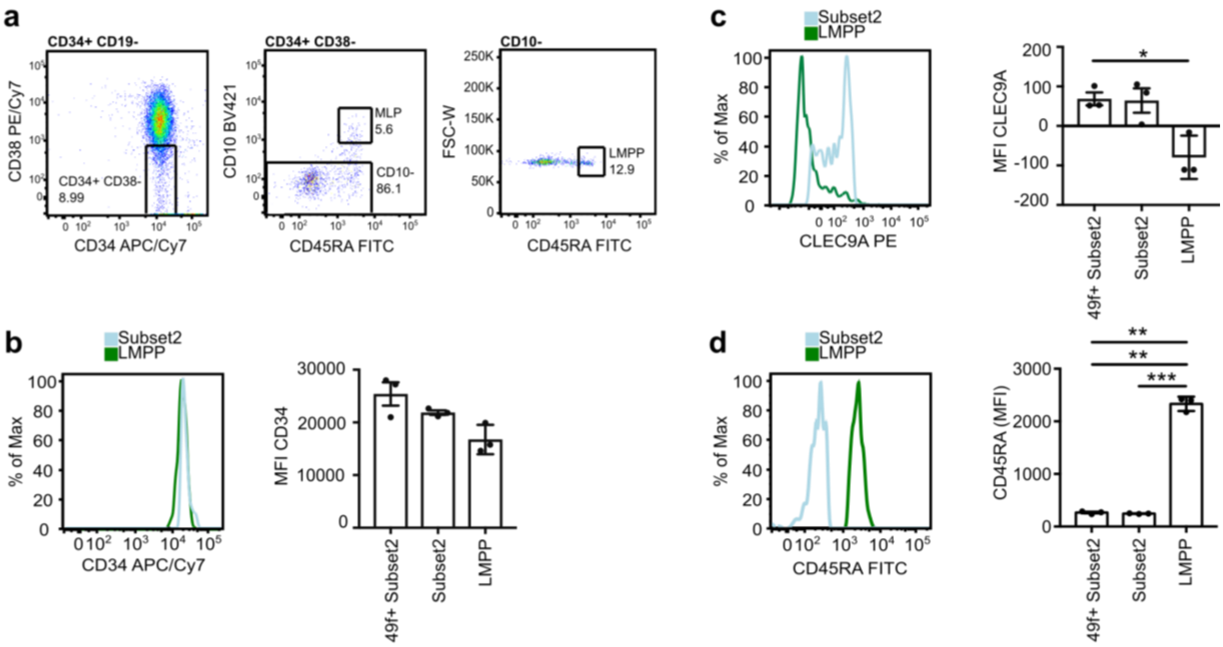

Supplementary Figure 7: Cell surface markers expression differences between LMPPs and Subset2 cells

a, Representative sorting strategy used to isolate LMPPs and MLPs progenitors at day 0 from $CD34^+$ CB cells. This strategy was used for experiments in Fig. 6. **b-d,** Cell surface marker expression comparison of CD34 (**b**), CLEC9A (**c**) and CD45RA (**d**) in $49f^+$ HSCs, Subset2 cells and LMPPs. A representative example of the expression of each marker in Subset2 cells and LMPPs is shown on the left. Bar graphs showing the Median Fluorescent Intensity (MFI) of each marker for $49f^+$ HSCs, Subset2 and LMPPs are shown on the right (n=3 independent CBs**,** mean ± SEM is shown, *p<0.05, **p<0.01, ***p<0.001 by one-way ANOVA with Tukey's multiple comparison.

Supplementary Table 1: *In vivo* limiting dilution assay of Subset1, Subset2, 49f^{+} Subset1 and 49f^{+} Subset2.

Weeks	Group	Dose	Engrafted/Injected Mice	Repopulating capacity			p-value
				lower	estimate	upper	
2	Subset 1	100	0/5	4334	1402	454	0.639
		500	3/9				
	Subset 2	100	0/5	2606	979	368	
		500	4/9				
8	Subset 1	100	1/3	780	296	113	0.077
		500	4/5				
	Subset 2	100	1/3	5209	1242	296	
		500	1/5				
20	Subset 1	50	4/8	1213	453	170	4.36×10^{-4}
		250	5/6				
		3000	10/11				
	Subset 2	50	0/4	6379	2684	1129	
		250	2/9				
		3000	4/7				
20	49f^{+} Subset 1	10	4/8	30.0	13.5	6.24	1.06×10^{-10}
		50	5/5				
		200	10/10				
	49f^{+} Subset 2	10	0/4	2143.1	685.5	219.5	
		50	1/9				
		200	2/9				

p-value was calculated via the ELDA method[3]

SUPPLEMENTARY REFERENCES

1. Notta, F. *et al.* Isolation of single human hematopoietic stem cells capable of long-term multilineage engraftment. *Science* **333,** 218–221 (2011).

2. Brennecke, P. *et al.* Accounting for technical noise in single-cell RNA-seq experiments. *Nat. Methods* **10,** 1093–1095 (2013).

3. Hu, Y. & Smyth, G. K. ELDA: Extreme limiting dilution analysis for comparing depleted and enriched populations in stem cell and other assays. *J. Immunol. Methods* **347,** 70–78 (2009).
