# Supplementary material for: Myelo-lymphoid lineage restriction occurs in the human haematopoietic stem cell compartment before lymphoid-primed multipotent progenitors
Source: Nat Commun. 2018 Oct 5;9:4100. doi: 10.1038/s41467-018-06442-4 (PMC6173731; doi:10.1038/s41467-018-06442-4)
Supplement: Supplementary file 3 — Description of Additional Supplementary Files [file 41467_2018_6442_MOESM3_ESM.pdf]

SUPPLEMENTARY DATA LEGENDS

Supplementary DATA 1: List of guide genes as determined by ICGS algorithm.

List of genes on which the heatmap shown in Fig. 3b is based.

Supplementary DATA 2: List of genes differentially expressed between $49f^+$ Subset1 and $49f^+$ Subset2.

Shown are genes with FDR <0.05 as determined by DESeq2. Of note, CD34 and CLEC9A expression were respectively 1.47 fold higher in $49f^+$ Subset2 and 1.9 fold higher in $49f^+$ Subset1, but with FDR >0.05.

Supplementary DATA 3: List of genes differentially expressed between Subset1 and Subset2.

Shown are genes with FDR <0.05 as determined by DESeq2. Of note, CD34 and CLEC9A expression were respectively 1.92 fold higher in Subset2 and 5.67 fold higher in Subset1.

Supplementary DATA 4: lineage composition of human grafts in mice transplanted with Subset1, Subset2, $49f^+$ Subset1 and $49f^+$ Subset2 cells.

Shown is the percentage of human engraftment and relative graft composition as percentage of My, Ly and Ery cells from engrafted mice at the indicated doses and weeks from the indicated groups.
